# Mechanism of soil microbial community degradation under long-term tomato monoculture in greenhouse

**DOI:** 10.3389/fmicb.2025.1587397

**Published:** 2025-07-29

**Authors:** Menglu Li, Xiaobo Chen, Yushuang Cui, Xin Yue, Lianfen Qi, Yali Huang, Changxiong Zhu

**Affiliations:** ^1^College of Environmental Science and Engineering, Hebei University of Science and Technology, Shijiazhuang, China; ^2^College of Food Science and Biology, Hebei University of Science and Technology, Shijiazhuang, China; ^3^Shijiazhuang Academy of Agriculture and Forestry Sciences, Shijiazhuang, China

**Keywords:** long-term continuous cropping, microbial community, Mantel test, potential function, soil deterioration

## Abstract

Tomato (*Solanum lycopersicum* L.), an economically significant crop, is frequently cultivated in greenhouses under continuous monoculture systems. Motivated by intensive agricultural practices and economic incentives, continuous cropping has become prevalent in China, yet it often results in soil degradation, including nutrient imbalances and microbial community shifts. However, the mechanisms driving soil deterioration in prolonged greenhouse monoculture remain unclear. In this study, soil samples from greenhouses with varying durations of continuous tomato cropping (1–3 years, 5–7 years, and >10 years) were analyzed for microbial and chemical parameters using 16S rRNA and ITS sequencing and soil nutrient assays. Results demonstrated a significant increase in fungal abundance and diversity in >10 years samples, alongside reduced bacterial richness. Co-occurrence network analysis revealed opposing trends in bacterial and fungal networks, indicating a shift from bacterial to fungal dominance. This shift correlated with impaired microbial functions, including diminished metabolic activity and impaired carbon-nitrogen cycling. PLS-PM model identified the accumulation of soil organic matter (SOM), nitrogen (N), and phosphorus (P) as key drivers of microbial community restructuring. Functional gene predictions based on 16S rRNA sequencing indicated that the expression of genes related to carbon (*tktA*/*tktB*, *accA*, *acsB*, *cooS*/*acsA*, *ppc*) and nitrogen (*pmoA-amoA*, *nxrA*, *hao*, *nasA*, *nasB*, *gdh*, *ureC*, *narG*, *nirB*, *nirK*, *norB*, *nosZ*) transformation were decreased. Mantel test further highlighted *KD4_96* and *Bacillus* as critical regulators of carbon and nitrogen dynamics. These findings elucidate mechanisms underlying soil degradation in long-term greenhouse monoculture systems and provide a theoretical basis for sustainable soil management strategies.

## 1 Introduction

China, the largest tomato producer of world, yields approximately 70 million tons annually (National Bureau of Statistics of China, 2023). Prolonged monoculture of tomatoes in greenhouses, defined as the repeated cultivation of a single crop without rotation or fallow periods, is widespread in northern China (Zhang and He, 2004; Chen et al., 2009). While economically advantageous, this practice often leads to replanting disease within 2 years and becomes widespread in greenhouses after 5 years of continuous cropping (Tan et al., 2021). Long-term continuous cropping has caused many problems, including acidification, salinization (Han et al., 2014), and microbial community imbalance (Zhao et al., 2019; Song et al., 2022; Dang et al., 2023).

Soil microbiota play pivotal roles in nutrient cycling, organic matter transformation, and ecosystem stability (Nunes et al., 2020; Li et al., 2021). However, intensive agricultural practices disrupt microbial dynamics, particularly in greenhouse environments characterized by high planting density, elevated temperature, humidity, and limited leaching (Karlen et al., 2019; Trivedi et al., 2020). Research has shown that soil parameters and plant community dynamics significantly influence microbial community structure and carbon (C) and nitrogen (N) cycling (Bertin et al., 2003; Chen et al., 2020; Ning et al., 2020; Shen W. et al., 2021; Liu et al., 2023). In greenhouse environments, conditions such as high planting density, elevated temperature and humidity, soil acidification, and limited leaching due to minimal rainfall further exacerbate changes in soil microflora.

Studies on various monoculture systems, including bananas and sweet potatoes, indicate that extended monoculture reduces bacterial diversity and promote harmful fungi, shifting the microflora from bacterial to fungal dominance (Sun et al., 2018; Gao et al., 2019). Similarly, research on tomato greenhouse monoculture has shown declines in bacterial diversity over time, accompanied by a significant rise in fungal abundance, often driven by the proliferation of soil-borne pathogens like *Fusarium* and *Aspergillus* (Ning et al., 2020; Zhao et al., 2023; Wei et al., 2024). This shift toward fungal dominance weakens soil ecosystem stability (Wei et al., 2024). Understanding the drivers of soil deterioration in monoculture systems is essential for developing effective soil management strategies.

Soil properties, such as pH, fertility, and organic matter content, strongly influence microbial community composition. For instance, research has shown that changes in soil pH, fertility, and organic matter partly drive microbial community shifts. In cucumber greenhouses, variations in bacterial communities are strongly associated with soil pH, available potassium (AK), total phosphorus (TP), and total potassium (TK), while fungal communities primarily respond to AK and TK (Chen et al., 2018; Karlen et al., 2019; Zhao et al., 2019; Xu et al., 2020; Dang et al., 2023). In another study of tomato continuous cropping for 20 years in North-west China, accumulated soil nutrients, including organic matter and nitrogen, were shown to drive changes in microbial communities, while it is surprised that no significant variations in fungal flora and function were found after long-term monocropping (Dang et al., 2023). However, in another study on continuous tomato cropping in northern China, accumulated soil nutrients, including organic matter, nitrogen and potassium, were shown to drive changes in microbial communities from “bacterial type” to “fungal type,” by reducing beneficial bacterial populations while increasing pathogenic fungi (Zhao et al., 2023). Research also suggests that soil chemical alterations in continuous monoculture weaken bacterial network stability, diminish metabolic functions, and enhance potential stress resistance functions (Dang et al., 2023). The microbial community would down-regulate the N-cycling gene abundances following organic fertilization to alleviate the soil N “crisis” under microbial N limitation during the three-year experimental period (Shen H. et al., 2021). While soil degradation in continuous monoculture greenhouse systems is a consistent finding, the specific effects of soil properties on microbial communities vary across studies and detailed research on the mechanisms underlying microbial community shifts in long-term greenhouse monoculture systems remains limited.

Hebei Province, a major vegetable-producing region in northern China, cultivates tomatoes across 3.24 × 10^4^ hectares of greenhouse systems (Shen, 2020). This study investigates microbial and chemical parameters in soils under varying durations of continuous tomato cropping (1–3, 5–7, and >10 years) in Langfang (the largest greenhouses tomato planting City of Hebei province). Objectives include: (1) assessing microbial community composition, co-occurrence networks, and functional shifts under prolonged monoculture; and (2) deciphering the main drivers of microbial and functional evolution. This study provides valuable insights into the soil degradation process, emphasizing the distinct responses of bacterial and fungal flora to long-term monoculture and offering theoretical guidance for the subsequent soil improvement in greenhouse.

## 2 Materials and methods

### 2.1 Sample collection and chemical properties assay

Soil samples were collected from greenhouses in Langfang City, Hebei Province (116°41′01″E, 39°32′18″N), characterized by a subhumid continental monsoon climate. The experience’s region has an annual sunshine duration of 2,740 h, an average temperature of 11.5°C, and annual precipitation of 540 mm.

Samples were sieved (2 mm), subdivided for microbial and chemical analyses, and stored at −80°C or air-dried, respectively. All sample sites followed a consistent cropping system, where tomato plants were uprooted after fruit harvest in May and replanted in autumn. Three sites per cropping duration (1–3a, 5–7a, 10a+) were sampled at 5–20 cm depth during the tomato flowering-fruiting period (March 2021). Within each site, five random sampling points were chosen, and soil samples were taken at a depth of 5–20 cm and approximately 20 cm from the tomato plants. All bulk soil samples were sieved through a 2-mm mesh after the removal of litter, stones, and soil earthworms and divided into two parts: one was stored at −80°C for microorganism analysis and the other was air-dried for soil chemical properties determination.

Soil chemical properties, including pH, electrical conductivity (EC), soil organic matter (SOM), total nitrogen (TN), total phosphorus (TP), total potassium (TK), alkaline hydrolysis nitrogen (AN), available phosphorus (AP), and available potassium (AK), were measured according to established protocols (Du et al., 2022; Dang et al., 2023).

### 2.2 DNA extraction, PCR amplification, and sequencing

Total microbial DNA was extracted from frozen soil samples using the OMEGA Soil DNA Kit (D5625-01; Omega Bio-Tek, Norcross, GA, United States). With the qualified soil DNA serving as the template and sterile water as the blank control, polymerase chain reaction (PCR) amplification was performed. The absence of bands on the gel image indicated no contamination during the amplification process. The target fragments for amplification were the V3–V4 region of the bacterial 16SrRNA gene and the ITS-V1 region of fungi. The upstream and downstream primers were 338F, 806R and 1737F, 2043R, respectively (Cui et al., 2025).

The PCR amplification system (25 μL) consisted of the following components: 5 μL of 5 × reaction buffer, 5 μL of 5 × GC buffer, 2 μL of dNTP at a concentration of 2.5 mmol L^−1^, 1 μL of forward primer at 10 μmol L^−1^, 1 μL of reverse primer at 10 μmol L^−1^, 2 μL of DNA template, 8.75 μL of double-distilled water (ddH₂O), and 0.25 μL of Q5 DNA Polymerase (5 U/μL).

The amplification conditions were as follows: initial denaturation at 98°C for 5 min, followed by 30 cycles of 98°C for 30 s (denaturation), 55°C for 30 s (annealing), and 72°C for 45 s (extension). After the cycling, a final extension was performed at 72°C for 5 min, and the reaction was terminated at 4°C.

The PCR amplification products were detected via 0.8% agarose gel electrophoresis. The qualified products were then entrusted to Shanghai Pansino Biotechnology Co., Ltd. for sequencing and microbial diversity analysis using the Illumina NovaSeq6000 platform. The datasets generated for this study can be found in the NCBI BioProject repository under accession number (PRJNA1273093).

### 2.3 Statistical analysis of data

The QIIME2 (2019.4) software was employed to carry out procedures such as primer trimming, quality filtering, denoising, sequence assembly, and chimera removal. Subsequently, the amplicon sequence variant (ASV) characteristic sequences and ASV tables were integrated. Regarding annotation, for the 16S ribosomal RNA (rRNA) gene of bacteria, the Greengenes database was utilized for annotation. For the internal transcribed spacer (ITS) sequences of fungi, the UNITE database was adopted for annotation purposes. A one-way analysis of variance (ANOVA), followed by the least significant difference (LSD) test at *p* < 0.05, was conducted using IBM SPSS Statistics 26 software to identify significant differences among treatments. The alpha diversity measures of soil fungal and bacterial populations were calculated by means of the “ggplot2” package within R. The microbial composition was investigated via principal coordinate analysis (PCoA) based on the Bray–Curtis distance (Bray and Curtis, 1957) using the “ape” package in R version. Co-occurrence network analysis was conducted by R and Gephi software to compare interaction complexities among bacterial and fungal taxa. The redundancy analysis (RDA) of the relationship between soil environmental factors and microbial community composition was conducted using Canoco 5.0 software, and corresponding graphs were generated. Functional predictions for bacteria and fungi were conducted using PICRUSt2 (Yuan et al., 2021), and associations between soil microbial metabolic functions and soil properties were evaluate by the Mantel test using ggplot2 in R software (version 4.1.3). PLS-PM is used to provide an intuitive graphical representation of the dynamic interaction between variables (Tenenhaus, 2008).

## 3 Results

### 3.1 Soil nutrient and enzyme index

With extended years of cropping, the contents of SOM, TN, TP, AN, and AP in the soil first decreased and then increased, reaching maximum values at the 10a+ samples. Compared to the 1–3a samples, SOM, TN, TP, AN, and AP of 10a+ samples increased by 61.51, 73.16, 107.72, 57.48, and 43.74%, respectively (*p* < 0.05). This indicates a substantial nutrient accumulation over time. However, soil pH remained relatively stable, fluctuating around 7.7, with no significant differences among the three cropping durations (Table 1). No significant differences were observed in the activities of soil enzymes (urease, alkaline phosphatase, sucrase, and catalase) among the three planting durations (Supplementary Table S1).

**Table 1:** Soil properties in different continuous planting years.

Indicators	1–3a	5–7a	10a+
pH	7.67 ± 0.21a	7.75 ± 0.14a	7.71 ± 0.10a
SOM (g/kg)	22.32 ± 2.28b	17.66 ± 3.70b	36.05 ± 2.80a
TN (g/kg)	1.90 ± 0.29b	1.61 ± 0.06b	3.29 ± 0.39a
TP (g/kg)	2.72 ± 0.47b	2.48 ± 0.09b	5.65 ± 0.50a
TK (g/kg)	30.67 ± 0.96ab	31.39 ± 0.85a	29.53 ± 1.03b
AN (mg/kg)	124.16 ± 18.52b	116.43 ± 18.98b	195.53 ± 9.65a
AP (mg/kg)	278.73 ± 75.99b	240.70 ± 29.46b	400.64 ± 63.29a
AK (mg/kg)	634.50 ± 122.26a	542.67 ± 21.94a	625.00 ± 142.31a

SOM, soil organic matter; TN, total nitrogen; TP, total phosphorus; TK, total potassium; AN, alkaline hydrolysis nitrogen; AP, available phosphorus; and AK, available potassium. The results were given as mean ± SD (standard deviation). Different letters followed by values show significant differences (*p* < 0.05) based on analysis of variance.

### 3.2 Number of ASVs

**Figure 1:**
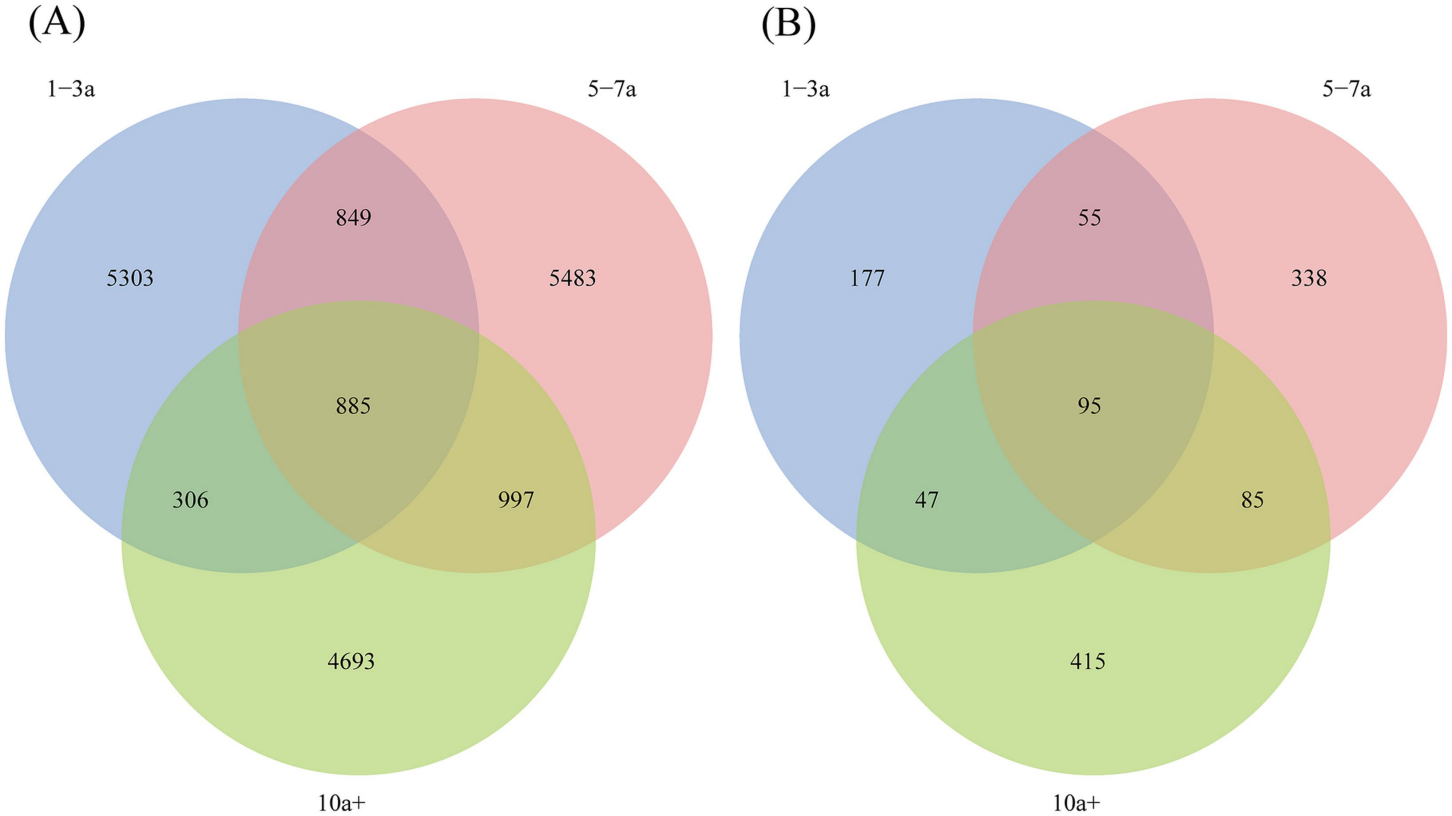
Venn diagram of soil bacterial **(A)** and fungal **(B)** at ASVs level under different planting years.

A total of 18,516 bacterial ASVs were identified from the nine soil samples, with only 885 shared ASVs, representing 4.78% of the total bacterial ASVs (Figure 1A). This indicates significant differences in bacterial community composition among the samples. In the 1–3a samples, 7,343 ASVs (39.66% of total bacterial ASVs) were identified, while the 5–7a and 10a+ samples contained 8,214 ASVs (44.36%) and 6,881 ASVs (37.16%), respectively. This suggests a slight decline in bacterial diversity with prolonged cropping duration.

For fungi (Figure 1B), a total of 1,212 ASVs were detected, with only 95 shared ASVs, accounting for 7.84% of the total fungal ASVs. In the 1–3a, 5–7a and 10a+ samples, fungal ASVs numbered 374 (30.86% of total fungal ASVs), 573 (47.28%), and 642 (52.97%), respectively, indicating a significant increase in fungal diversity with extended cropping years.

### 3.3 Alpha and beta diversity

The alpha diversity of bacterial communities exhibited a temporal decline, though no significant differences (*p* > 0.05) were observed among the three cropping durations. Notably, the Chao1 index, observed species richness, and Shannon index reached their highest values in the 5–7a samples (Table 2). In contrast, fungal community diversity, as indicated by the Chao1 index and observed species richness, differed significantly across cropping durations (*p* < 0.05). Compared to the 1–3a samples, these indices increased by 58.45 and 58.18% in the 5–7a and 10a+ samples, respectively. While Pielou’s evenness, Shannon, and Simpson indices showed no significant differences (*p* > 0.05), fungal diversity indices demonstrated an overall increasing trend with prolonged cropping.

**Table 2:** Soil alpha diversity index under different continuous planting years.

Microbiome	Samples	Chao1	Observed_species	Pielou_e	Shannon	Simpson
Bacteria	1–3a	3738.97 ± 307.2a	3581.70 ± 225.99a	0.90 ± 0.01a	10.62 ± 0.03a	0.99 ± 0.01a
	5–7a	3937.83 ± 94.99a	3690.20 ± 155.00a	0.90 ± 0.01a	10.71 ± 0.15a	0.99 ± 0.01a
	10a+	3471.43 ± 82.67a	3269.75 ± 53.95a	0.90 ± 0.01a	10.47 ± 0.07a	0.99 ± 0.01a
Fungi	1–3a	220.61 ± 43.02b	220.45 ± 43.20b	0.50 ± 0.19a	3.92 ± 1.61a	0.77 ± 0.24a
	5–7a	322.85 ± 37.85ab	321.60 ± 36.35ab	0.62 ± 0.01a	5.19 ± 0.19a	0.93 ± 0.01a
	10a+	349.55 ± 7.11a	348.70 ± 7.07a	0.64 ± 0.01a	5.37 ± 0.05a	0.92 ± 0.01a

The results were given as mean ± SD (standard deviation). Different letters followed by values show significant differences (*p* < 0.05) based on analysis of variance.

**Figure 2:**
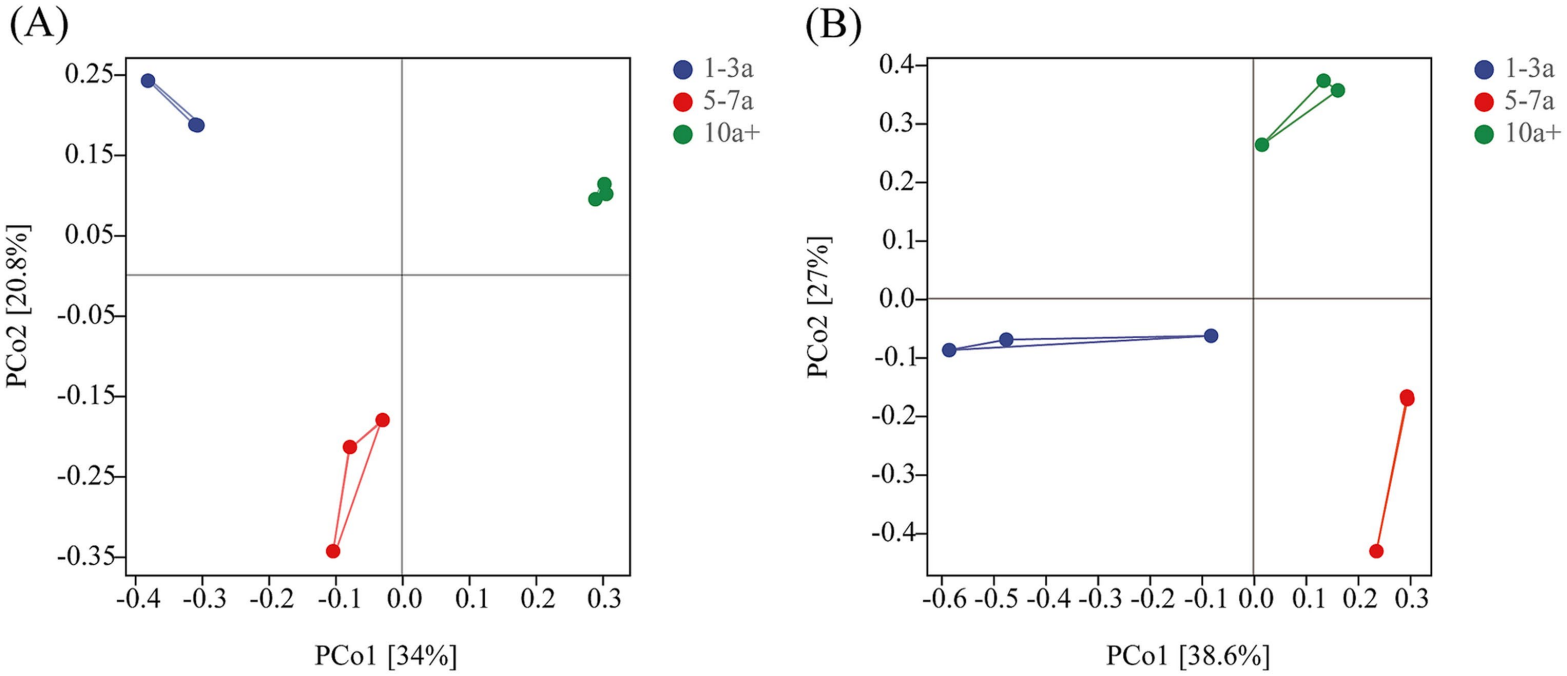
Principal co-ordinates analysis of bacterial **(A)** and fungal **(B)** communities under different continuous planting years.

Principal coordinate analysis (PCoA) revealed distinct clustering patterns in both bacterial (Figure 2A) and fungal (Figure 2B) communities across cropping durations. The first two PCoA axes explained 54.8 and 65.6% of the total variance for bacterial and fungal communities, respectively.

### 3.4 Composition of microbial community

**Figure 3:**
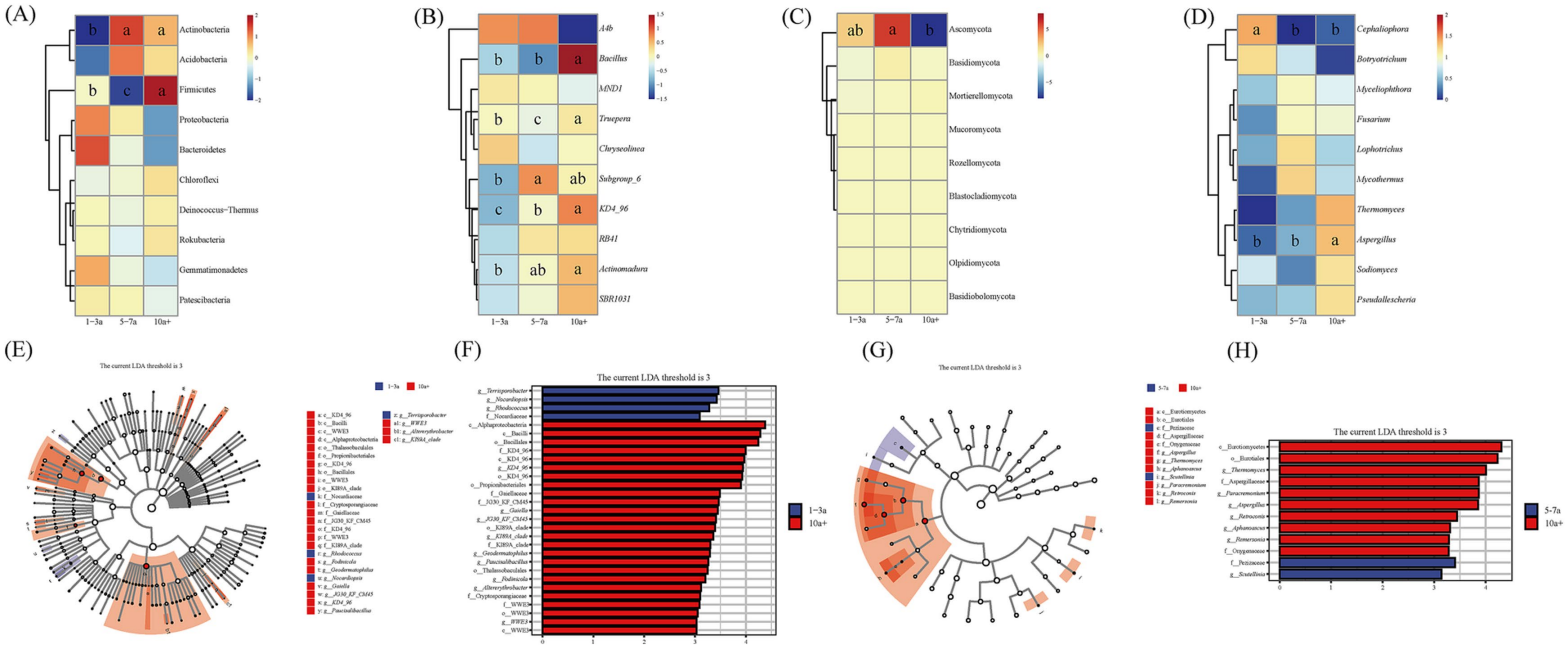
Bacterial **(A,B)** and fungal **(C,D)** community composition at phyla and genus levels, respectively, under different continuous planting years. Taxonomic cladograms of linear discriminant analysis effect size (LEfSe) analysis of tomato at three different continuous planting years, depicting ASVs with absolute linear discriminant analysis (LDA) scores larger than 3.0. **(E,F)** Biomarkers of soil bacteria from different continuous planting years of tomato. **(G,H)** Biomarkers of soil fungi from different continuous planting years of tomato.

The top 10 dominant bacterial phyla remained consistent across cropping durations, with their relative abundances ranked from highest to lowest in Figure 3A. Proteobacteria, Bacteroidota, and Gemmatimonadota exhibited a decreasing trend with prolonged cropping, whereas Actinobacteriota, Chloroflexi, and Firmicutes increased significantly. Specifically, the relative abundance of Actinobacteriota in the 5–7a and 10a+ samples increased by 20.09 and 15.96%, respectively, compared to the 1–3a samples. Firmicutes reached its peak abundance (7.38%) in the 10a+ samples (*p* < 0.05) (Figure 3A). Similarly, the top 10 dominant bacterial genera remained consistent across cropping durations, with abundances ordered from highest to lowest (Figure 3B). Genera such as *Subgroup_6*, *Truepera*, *Bacillus*, *RB41*, *Actinomadura*, *SBR1031*, and *KD4_96* showed increased abundance over time, while *A4b* and *MND1* declined significantly (*p* < 0.05) (Figure 3B).

Among fungi, the top five dominant phyla were Ascomycota, Basidiomycota, Mortierellomycota, Mucoromycota, and Rozellomycota. Ascomycota dominated across all cropping durations, with relative abundances of 76.33, 90.35, and 75.91% in the 1–3a, 5–7a, and 10a+ samples, respectively (Figure 3C). Basidiomycota followed a similar trend, showing abundances of 0.18, 1.28, and 0.48% in the same order. Mortierellomycota (0.22, 0.63, 0.98%) and Mucoromycota (0.01, 0.05, 0.15%; *p* < 0.05) exhibited progressive increases with prolonged cropping.

The top five fungal genera remained unchanged across durations (Figure 3D). *Cephaliophora* abundance decreased significantly over time, while *Myceliophthora*, *Fusarium*, and *Lophotrichus* peaked in the 5–7a samples before declining in the 10a+ samples. Notably, *Aspergillus* increased from 0.24% in 1–3a to 1.69% in 10a+ samples, demonstrating a significant upward trend (*p* < 0.05) (Figure 3D).

LEfSe linear discriminant analysis (LDA >3.0) identified key biomarker taxa for bacterial and fungal communities across cropping durations (Figures 3E–H). For bacteria, 29 taxa were significantly enriched: *g__Terrisporobacter*, *g__Nocardiopsis*, and *g__Rhodococcus* were primary biomarkers in 1–3a samples, whereas c__Bacilli, o__Bacillales, and taxa affiliated with the *KD4_96* lineage (o__KD4_96, *g__KD4_96*, c__KD4_96, f__KD4_96) dominated in 10a+ samples. For fungi, 12 taxa were enriched, with f__Pezizaceae and c__Eurotiomycetes serving as biomarkers for 5–7a and 10a+ samples, respectively.

### 3.5 Co-occurrence networks

**Figure 4:**
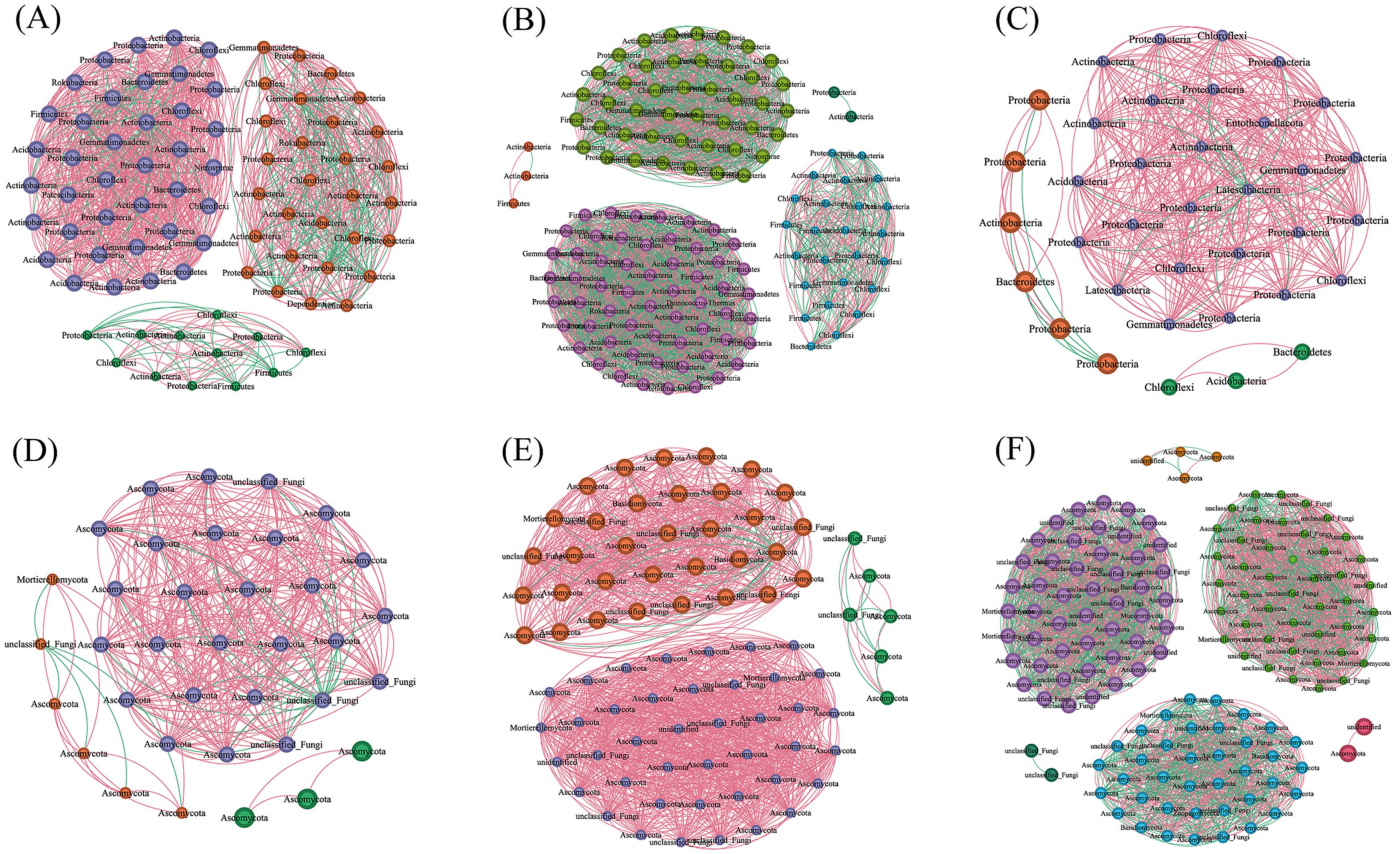
Analysis of soil microbial community interaction network under different continuous planting years. **(A–C)** Bacterial co-occurrence networks in 1–3a, 5–7a and 10a+, respectively. **(D–F)** Fungal co-occurrence networks in 1–3a, 5–7a and 10a+, respectively. The red and green lines indicated positive and negative interactions, respectively, between two individual nodes. The co-occurring networks are colored by module.

Distinct shifts in bacterial and fungal co-occurrence networks were observed with prolonged monoculture practices (Table 3 and Figure 4). Compared to samples from 1–3 years of cultivation, both bacterial and fungal networks exhibited similar trends in 5–7a samples, characterized by increased node numbers, edge counts, and network density. However, divergent patterns emerged in 10a+ samples. For bacterial networks, key parameters including node count, edge connections, average degree, and modularity decreased significantly compared to 1–3a samples, whereas network density rose, suggesting tighter interactions among taxa. Conversely, fungal networks maintained an upward trend in 10a+ samples, with all parameters continuing to increase beyond levels observed in 5–7a samples. These contrasting dynamics highlight opposing temporal trends between bacterial and fungal networks under extended monoculture system, as visually shown in Figure 4.

**Table 3:** Topological properties of molecular ecological network under different continuous planting years.

Topological properties	Bacteria			Fungi		
	1–3a	5–7a	10a+	1–3a	5–7a	10a+
Number of node	79	132	36	36	80	139
Number of edge	1,175	2,903	369	369	1,363	2,829
Average degree	29.747	43.985	20.500	20.500	34.075	40.705
Modularity	0.519	0.559	0.093	0.093	0.488	0.657
Network density	0.381	0.336	0.586	0.586	0.431	0.295

### 3.6 Prediction of microbial functions

**Figure 5:**
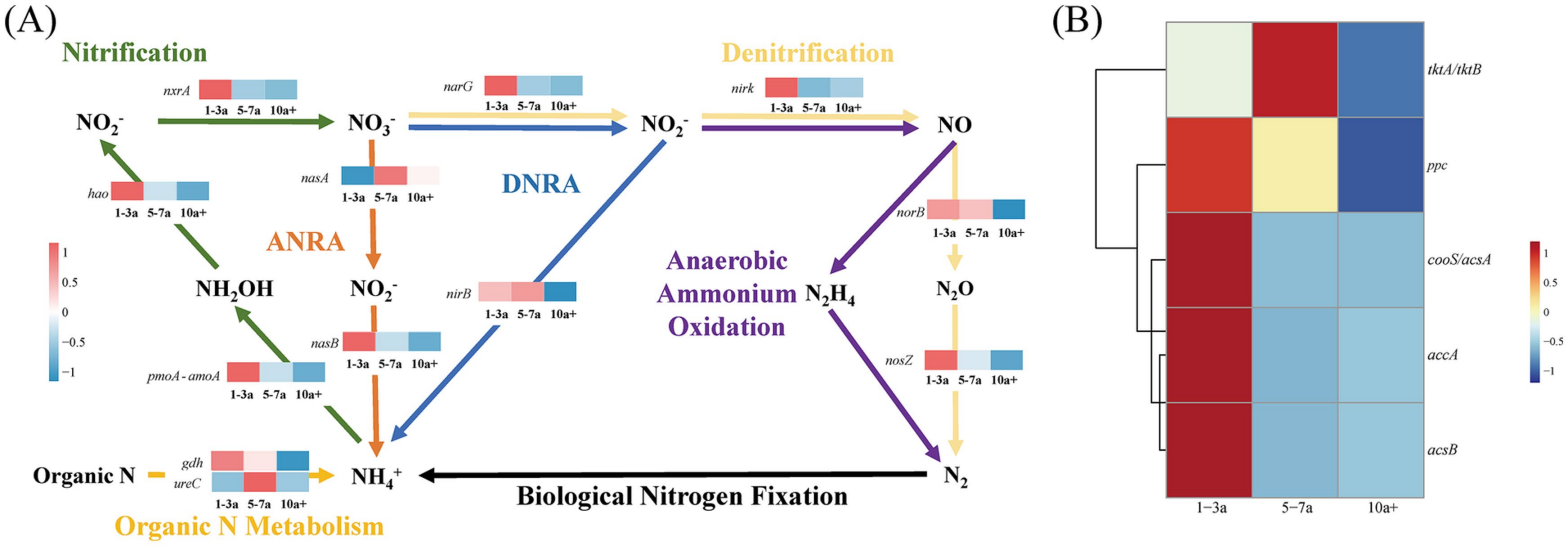
Variations in the expression of the key nitrogen transformation functional genes **(A)** and carbon fixation functional genes **(B)** within the soil microbial community under diverse continuous cropping durations.

Functional gene dynamics in carbon and nitrogen cycling were analyzed to assess microbial metabolic potential under prolonged greenhouse monoculture using PICRUSt2-based predictions. A total of 17 differentially abundant genes were identified in the process of long-term greenhouse monoculture, comprising 12 nitrogen-cycling-related genes and 5 carbon-cycling-related genes (Figure 5 and Table 4).

**Table 4:** Carbon fixation genes under different continuous cropping years.

Pathway	Energy source	Environment	Key genes
Calvin–Benson–Bassham cycle	Solar	Aerobic	*tktA*/*tktB*
3-Hydroxypropionate/4-Hydroxybutylate cycle	Solar	Aerobic	*accA*
Reductive acetyl-CoA cycle	Hydrogen	Anaerobic	*acsB* *cooS*/*acsA*
Dicarboxylate/4-hydroxybutyrate cycle	Hydrogen	Anaerobic	*ppc*

The transcriptional activity of these genes was closely linked to shifts in the core bacterial community structure during continuous tomato cultivation. In nitrogen cycling pathways, the relative abundance of 10 genes associated with denitrification (*narG*, *nirK*, *norB*, *nosZ*), nitrification (*pmoA-amoA*, *hao*, *nxrA*), dissimilatory nitrate reduction (*narG*, *nirB*), and organic N metabolism (*gdh*, *ureC*) declined significantly in 10a+ samples compared to 1–3a samples. Notably, only *nasA* (assimilatory nitrate reduction) exhibited increased abundance in long-term monoculture systems (Figure 5). Concurrently, carbon-cycling gene expression displayed marked suppression, with *tktA*/*tktB* (transketolase), *accA* (acetyl-CoA carboxylase carboxyl transferase), *acsB* (acetyl-CoA synthase), *cooS*/*acsA* (carbon monoxide dehydrogenase), and *ppc* (phosphoenolpyruvate carboxylase) showing reductions of 1.74, 45.19, 76.86, 50.24 and 11.99%, respectively, in 10a+ samples (Table 4). These systematic declines in functional gene abundance suggest a progressive attenuation of microbial contributions to biogeochemical cycling as monoculture duration increases.

### 3.7 Relationships between soil properties and microbial traits

**Figure 6:**
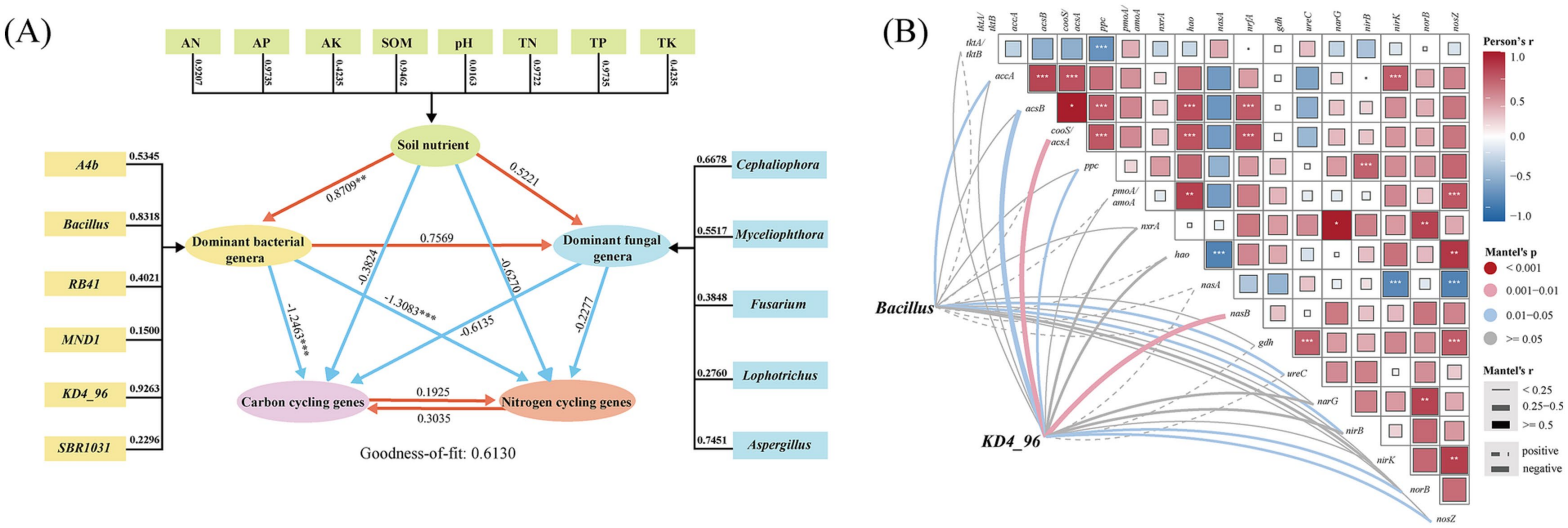
Partial least squares model of soil microbial communities, soil nutrients, soil carbon (C) and nitrogen (N) cycling genes in soil **(A)**. Mantel tests of associations between soil factor and microbial metabolic functional genes **(B)**. Functional genes of carbon and nitrogen transformation (based on PICRUST2) related to microbial community composition by partial Mantel tests.

Soil nutrient parameters exhibited variable contributions to explaining microbial community structure (Figure 6A), with available phosphorus (AP; weight = 0.9735) and total phosphorus (TP; weight = 0.9735) demonstrating the highest explanatory power. In contrast, pH (0.0163), available potassium (AK; 0.4235), and total potassium (TK; 0.4235) showed minimal influence. Among dominant bacterial taxa, *Bacillus* (weight = 0.8318) and *KD4_96* (0.9262) emerged as key contributors to soil microbial community dynamics. Pathway analysis further revealed a strong negative correlation between soil nutrient levels and dominant bacterial genera on one hand, and carbon-nitrogen (C/N) cycling genes on the other. In contrast, a positive correlation was observed between soil nutrient levels and dominant fungal genera. Notably, the direct effects of bacterial taxa on genes involved in soil carbon and nitrogen cycling were the most pronounced (path coefficients = −1.2463 and −1.3083).

LEfSe analysis and partial least squares path modeling (PLS-PM) identified *Bacillus* and *KD4_96* as keystone taxa, each exhibiting distinct gene associations. To elucidate their functional roles in C/N cycling under soil degradation, Mantel tests (Figure 6B) were performed. *Bacillus* displayed significant negative correlations (*p* < 0.05) with three genes: *accA* (acetyl-CoA carboxylase carboxyl transferase, carbon fixation), *ureC* (urease, organic N metabolism), and *nirB* (nitrite reductase, dissimilatory nitrate reduction). In contrast, *KD4_96* showed stronger negative associations (*p* < 0.01) with *acsB* (acetyl-CoA synthase, carbon fixation), *cooS*/*acsA* (carbon monoxide dehydrogenase), *nasB* (assimilatory nitrate reductase, carbon fixation), and moderate correlations (*p* < 0.05) with denitrification genes *norB* (nitric oxide reductase, denitrification) and *nosZ* (nitrous-oxide reductase, denitrification). These antagonistic relationships suggest taxon-specific regulatory effects on biogeochemical processes, with both genera broadly suppressing C/N transformation pathways in long-term monoculture systems, highlighting the strong influence of soil microbial communities on these processes.

## 4 Discussion

### 4.1 Imbalance of microbial communities under long-term monocropping

Accumulating evidence indicates that prolonged monocropping drives a functional transition in soil microbial communities from bacterial to fungal dominance (Liu et al., 2020; Chen J. et al., 2022; Chen Y. et al., 2022; Wu et al., 2022). Our findings corroborate this trend, demonstrating a progressive decline in bacterial amplicon sequence variants (ASVs) alongside a marked increase in fungal ASVs over time (Figure 1). Fungal richness and diversity reached their highest levels after 10 years of monoculture, whereas bacterial alpha diversity showed minimal variation across cropping years (Table 2), aligning with prior observations (Yang et al., 2020). This bacterial-to-fungal shift mirrors broader ecological patterns observed in degraded agroecosystems, where simplified cropping systems favor fungal proliferation (Jin et al., 2022; Wei et al., 2023; Xie et al., 2023).

Changes in microbial taxa abundance were also observed with extended monoculture. Key phyla associated with nutrient cycling (e.g., Proteobacteria, Bacteroidota, Gemmatimonadota) and disease suppression (Ascomycota) exhibited declining relative abundances, potentially compromising soil multifunctionality (Wang et al., 2023; Wei et al., 2023). Conversely, stress-tolerant taxa such as Actinobacteriota and Firmicutes—known for their metabolic versatility and sporulation capacity—increased significantly (Palaniyandi et al., 2013; Wu et al., 2019; Xu et al., 2020; Alami et al., 2021). In soils with long-term monocropping, an elevation in the abundance of Firmicutes is commonly accompanied by a decline of the abundance of Proteobacteria (Wei and Yu, 2018), consistent with our results. The abundance of Firmicutes has been shown to increase under stressed soil conditions, likely reflecting adaptive strategies under nutrient-depleted and pathogen-enriched conditions (Stephens, 1998; Xu et al., 2020). In addition to *Bacillus* and *Actinomadura*, we also noticed that the abundance of some other special genera changed correspondingly with soil quality degradation (Figure 4). Enrichment of *RB41* (polycyclic aromatic hydrocarbon degradation) (Shen et al., 2018) and *KD4_96* (organic decomposition and pathogen antagonism) (Li H. et al., 2022; Li M. et al., 2022) suggests microbial adaptation to organic pollutant stress in greenhouse soils. Conversely, declines in beneficial taxa—including *A4b* (tomato rhizosphere symbiont) (Wei et al., 2024) and *Cephaliophora* (pathogen suppression and pollutant degradation) (Zlotnikov et al., 2007)—may impair plant resilience. Notably, the proliferation of phytopathogenic fungi (*Fusarium*, *Aspergillus*) and the bacterial genus *SBR1031* (a facilitator of *Ralstonia solanacearum* virulence) (Huang et al., 2019) highlights escalating disease risks under long-term monoculture.

### 4.2 Divergent responses of bacterial and fungal networks in monoculture

Soil microbial co-occurrence networks are critically shaped by edaphic factors, with soil physicochemical properties exerting dominant selective pressures (Huang et al., 2023). Our study revealed divergent trajectories in bacterial and fungal network architectures under prolonged monoculture (10a+), reflecting a systemic transition toward fungal-dominated community interactions. In 10a+ soils, fungal networks exhibited elevated node counts, edge densities, average degrees, and modularity—indicators of expanded scale, structural complexity, and functional redundancy of the fungal network. Conversely, bacterial networks displayed marked declines in these parameters, suggesting fragmentation of interspecies connectivity and reduced niche partitioning.

Notably, 5–7 years’ short-term monoculture elicited concordant enhancements in both bacterial and fungal networks, with significant increases in node/edge counts, average degrees, and modularity. This transient phase likely reflects adaptive community restructuring in response to nutrient enrichment (e.g., organic matter accumulation), which initially supports microbial cooperation and metabolic diversification. However, prolonged nutrient oversaturation in 10a+ systems appears to destabilize bacterial consortia, potentially through competitive exclusion or metabolic niche overlap, while favoring fungal taxa adapted to high-stress environments. Such dichotomy aligns with ecological theory which positing that fungi, with their hyphal networks and enzymatic versatility (Morrissey et al., 2023).

### 4.3 Functional shifts in microbial activity with community imbalance

Soil microbial functionality is intrinsically linked to community composition and structural dynamics (Lu et al., 2022), a relationship well-documented in agroecosystems (Saraf et al., 2014; Shi et al., 2024). In this study, prolonged tomato monoculture significantly impaired nitrogen (N) cycling by suppressing the abundance of functional genes associated with N₂ fixation, denitrification, dissimilatory nitrate reduction to ammonium (DNRA), ammonification, and assimilatory N reduction. This down regulation of N-cycling genes likely reflects microbial adaptation to alleviate N limitation—a “soil N crisis”—arising from prolonged nutrient depletion over a decade of monocropping. Notably, microbial nutrient limitation exerted selective pressure on key N-transformation pathways, particularly nitrification and denitrification, as evidenced by reduced gene expression.

PLS-PM and Mantel tests identified accumulated soil nutrients—including SOM, TN, TP, AN, and AP—as primary drivers of microbial community restructuring. While these nutrients typically support microbial proliferation (Jin et al., 2022; Qin et al., 2024), their over saturation in monoculture systems favored stress-tolerant taxa (e.g., *SBR1031* and *KD4_96*) while suppressing beneficial genera such as *A4b* (rhizosphere symbiont) and *Cephaliophora* (pathogen antagonist). The result of RDA (Supplementary Figure S1) corroborated these shifts, revealing strong correlations between dominant microbial taxa and nutrient levels in 10a + soils. These findings suggest that nutrient accumulation disrupts microbial equilibrium by enriching taxa adapted to high-resource, high-stress environments. Taxonomic responses further highlighted ecological trade-offs. Species declining in abundance (e.g., network stabilizers negatively correlated with N accumulation) were replaced by taxa indicative of community destabilization (Ren et al., 2021; Zhao et al., 2021). For instance, key species *KD4_96* and *Bacillus* (Figures 6A,B), whose abundance increased under monoculture, has been as an indicator of soil quality deterioration and associated with soil microbial community instability (Ren et al., 2021; Xun et al., 2021). Together, these results suggest that a competitive, instability-prone communities developed over a 10-year monoculture period in this study.

Mantel tests integrating these results demonstrated that long-term monoculture degraded bacterial co-occurrence networks and weakened C/N transformation capacity, whereas fungal communities exhibited structural and functional reinforcement. This dichotomy underscores a systemic shift from bacterial to fungal dominance in soils subjected to extended monocropping. In the case of soils subjected to long-term monoculture, the application of organic fertilizers or bio-organic fertilizers can mitigate excessive nutrient enrichment and restore microbial diversity (Ahsan et al., 2023). Future research could conduct targeted regulatory measures by identifying the missing and enriched microbial taxa to resolve the issue of soil micro-ecological imbalance. Additionally, it is essential to use large size sample to enhance the generalizability of the research findings (Li et al., 2019; Tan et al., 2021).

## 5 Conclusion

This study demonstrates that long-term tomato monoculture in greenhouses induces significant shifts in microbial communities, characterized by reduced bacterial diversity and increased fungal dominance. Co-occurrence network analysis and functional predictions reveal the deterioration of microbial interactions and nutrient cycling capacities over time. The accumulation of soil organic matter (SOM), nitrogen (N), and phosphorus (P) emerged as primary drivers of these changes. These findings emphasize the importance of adopting sustainable soil management practices to mitigate the adverse effects of prolonged monoculture and preserve soil health in greenhouse systems.

## Data Availability

The original contributions presented in the study are publicly available. This data can be found here: https://www.ncbi.nlm.nih.gov, accession number PRJNA1273093.
