# Supplementary material for: Mechanism of soil microbial community degradation under long-term tomato monoculture in greenhouse
Source: Front Microbiol. 2025 Jul 29;16:1587397. doi: 10.3389/fmicb.2025.1587397 (PMC12339526; doi:10.3389/fmicb.2025.1587397)
Supplement: Supplementary file 1 [file Supplementary_file_1.docx]

Supplementary Material

1. **Supplementary Figures and Tables**

## 1.1 Supplementary Figure


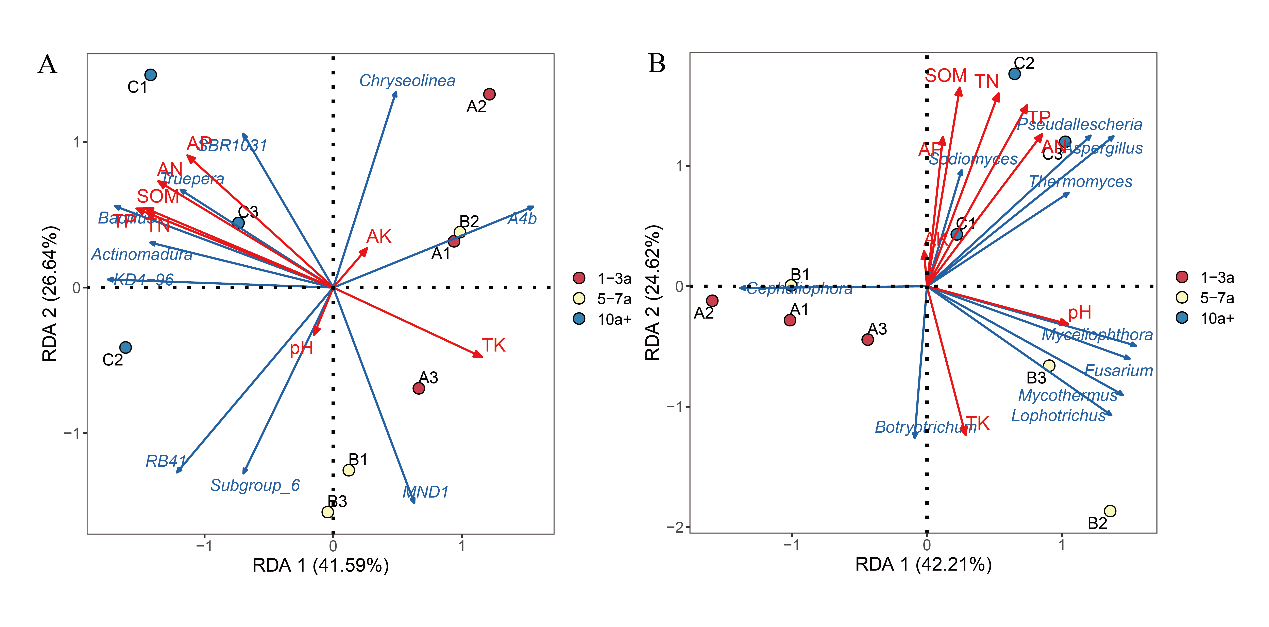
 **Supplementary Figure S1.** Correlations between planting years, microbial taxa composition, metabolic functions, and soil environmental factors. RDA analysis of relationship of soil environmental factors and bacterial (A) and fungal (B) community.

## 1.2 Supplementary Table

**Table S1** Soil enzyme activities across different durations of continuous cropping

|  | UE/U·g^−1^ | ALP/nmol·(h·g)^−1^ | CAT/umol(h·g)^−1^ | SC/mg(d·g)^−1^ |
| --- | --- | --- | --- | --- |
| 1-3a | 1693.33±167.05a | 71.16±0.39b | 8.16±0.24a | 4.70±0.49a |
| 5-7a | 1543.92±268.14a | 72.13±0.67a | 8.23±0.20a | 4.25±0.93a |
| 10a+ | 1613.85±257.54a | 71.77±0.66ab | 8.20±0.11a | 4.35±0.39a |

UE: Urease; ALP: Alkaline phosphatase; CAT: Catalase; SC: Sucrase. The results were given as mean ± SD (standard deviation). Different letters followed by values show significant differences (p < 0.05) based on analysis of variance.
